# Association between the overall burden of comorbidity and Ct values among the older patients with Omicron infection: Mediated by inflammation

**DOI:** 10.3389/fimmu.2023.1145044

**Published:** 2023-03-14

**Authors:** Meixia Wang, Hongfei Mi, Na Li, Qingfeng Shi, Wei Sun, Tingjuan He, Jiabing Lin, Wenting Jin, Xiaodong Gao, Bijie Hu, Chenghao Su, Jue Pan

**Affiliations:** ^1^ Department of Hospital Infection Management, Zhongshan Hospital, Fudan University (Xiamen Branch), Xiamen, Fujian, China; ^2^ Department of Infectious Diseases, Zhongshan Hospital, Fudan University, Shanghai, China; ^3^ Department of Hospital Infection Management, Zhongshan Hospital, Fudan University, Shanghai, China

**Keywords:** CT values, SARS-CoV-2, the elderly, inflammation, mediation effect, comorbidity burden

## Abstract

**Objectives:**

To investigate the associations between the overall burden of comorbidity, inflammatory indicators in plasma and Ct values among the elderly with COVID-19.

**Methods:**

We conducted a retrospective observational study. The results of each nucleic acid test of during hospitalization were obtained. Linear regression models assessed the associations between the overall burden of comorbidity, inflammatory indicators in plasma and Ct values among the elderly. A causal mediation analysis was performed to assess the mediation effects of inflammatory indicators on the association between the overall burden of comorbidity and Ct values.

**Results:**

A total of 767 COVID-19 patients aged ≥ 60 years were included between April 2022 and May 2022. Patients with a high burden of comorbidity had significantly lower Ct values of the ORF gene than subjects with a low burden of comorbidity (median, 24.81 VS 26.58, *P* < 0.05). Linear regression models showed that a high burden of comorbidity was significantly associated with higher inflammatory responses, including white blood cell count, neutrophil count and C-reactive protein. Also, white blood cell count, neutrophil count, C-reactive protein and the overall burden of comorbidity assessed by age-adjusted Charlson comorbidity index were independent risk factors for the Ct values. A mediation analysis detected the mediation effect of white blood cells on the association between the burden of comorbidity and Ct values, with the indirect effect estimates of 0.381 (95% CI: 0.166, 0.632, *P* < 0.001). Similarly, the indirect effect of C-reactive protein was -0.307 (95% CI: -0.645, -0.064, *P* = 0.034). White blood cells and C-reactive protein significantly mediated the relationship between the burden of comorbidity and Ct values by 29.56% and 18.13% of the total effect size, respectively.

**Conclusions:**

Inflammation mediated the association between the overall burden of comorbidity and Ct values among elderly with COVID-19, which suggests that combined immunomodulatory therapies could reduce the Ct values for such patients with a high burden of comorbidity.

## 1 Introduction

The Omicron variant has spread rapidly worldwide, inducing a pandemic in Shanghai in March 2022 (1). Thus far, at least 32 mutations have been identified in the spike protein, resulting in high transmissibility and immune escape (2–4). On the other hand, Omicron infection was associated with a lower rate of hospital admission and mortality (5, 6). However, the elderly were more likely to be infected with Omicron due to immunosenescence and high comorbidity burden (7, 8). The rate of severe Omicron infections were increased in the older patients and in individuals with comorbidities such as hypertension, diabetes, cardiovascular disease, and chronic respiratory disease (9, 10). Moreover, it was reported that the severe illness was related to high viral copies (11, 12). Therefore, understanding the underlying mechanism of Ct values, representing the degree of SARS-CoV-2 viral loads among the elderly with COVID-19, may be helpful for the early implementation of therapy.

As we know, viral loads are associated with infectiousness, transmissibility, disease severity, and mortality (11). Previous studies reported that the elderly and chronic medical diseases might influence the viral loads/Ct values. Patients’ age was found to be positively correlated with the viral loads (13, 14), partly because of immunosenescence (15). Additionally, the elderly were more likely to have comorbidities. Several studies have identified that congestive heart failure, hypertension, diabetes, chronic kidney disease, and coronary artery disease are associated with higher SARS-CoV-2 copies of the viral genome or lower Ct values (9, 16, 17). Most of these studies mainly focused on the simple relationship between a single chronic medical disease and Ct values/viral loads. However, a person might suffer a high burden of comorbidity with multiple chronic medical diseases. The overall comorbidity burden is of important as it considers multiple chronic medical conditions. The effect of the overall burden of comorbidity on Ct values remains unclear and needs to be further elucidated.

The inflammatory response has recently emerged as an essential factor in COVID-19 patients. Numerous studies reported a direct association between preexisting comorbidities and inflammation, which might impact the immune response to COVID-19. For example, the experimental model showed that COVID-19 with elevated glucose levels directly promoted viral replication, cytokine production, and subsequent T cell dysfunction (18, 19). It was also reported that COVID-19 with hypertension delayed viral clearance and exacerbated airway hyper inflammation (20, 21). Cancer patients with COVID-19 have impaired lymphocyte function, neutropenia, and decreased in white cell count (22, 23). This suggests that the delayed viral clearance and hyper inflammation are involved in COVID-19 patients with comorbidities, possibly contributing to severe illness. Furthermore, the correlation of respiratory viral loads were found to be correlated with inflammatory indicators in the plasma of elderly patients (24). White blood cells, neutrophils, and lymphocytes were significantly lower in patients with a high viral load (Ct ≤ 25) (25). Considering these results, the associations between Ct values and the preexisting comorbidities might be affected by inflammation. However, most previous the epidemiological reports focused on evaluating the simple association between comorbidity and Ct values. The effect of inflammation on the relationship between preexisting comorbidities and Ct values needs to be further elucidated in real-world data. Due to the importance of viral loads in disease severity, assessing the effect of inflammation on the association between Ct values and comorbidities might inform proper therapeutic strategies, especially for the elderly with comorbidities.

The aim of this study was to investigate the associations between the overall burden of comorbidity, inflammatory indicators in plasma, and Ct values among the elderly with COVID-19. A mediation analysis was conducted to explore the mediation effects of inflammatory indicators on the relationship between the overall burden of comorbidity and Ct values.

## 2 Methods

### 2.1 Study population

This retrospective observational study was conducted at Zhongshan Hospital, Fudan University (Geriatrics Medicine Center), between April 2022 and May 2022. The Geriatrics Center was designated as a temporary COVID-19 hospital during the Omicron outbreak in Shanghai, mainly receiving older patients. Patients with positive nucleic acid testing for SARS-CoV-2 were included. Only COVID-19 patients aged ≥60 years old were eligible in this study. Patients without complete medical history were excluded from this study. Finally, 767 COVID-19 patients were included. There demographic information (age, gender, COVID-19 vaccination, time of hospital admission and discharge), COVID-19 diagnosis, chronic medical conditions, laboratory results of inflammatory indicators in plasma before treatment (white blood cell, neutrophil count, lymphocyte count, C-reactive protein and procalcitonin), and the results of each nucleic acid test of during hospitalization (Ct values) were retrospectively collected. This study was approved by the Ethical Committee of Zhongshan Hospital, Fudan University.

Patients were divided into a non-severe group and a severe group according to disease severity. Patients were allocated to the severe group if they satisfied any of the following requirements proposed by the Diagnosis and Treatment of New Coronavirus Pneumonia (ninth edition):1) shortness of breath, RR≥30 times/min; 2) in the resting state, oxygen saturation ≤93% during air inhalation; 3) arterial partial pressure of oxygen (PaO2)/inspired oxygen concentration (FiO2) ≤300 MMHG (1mmHg=0.133kPa); 4) the clinical symptoms were progressively aggravated, and the chest imaging showed that the lesions significantly progressed > 50% within 24-48 hours; 5) respiratory failure requiring mechanical ventilation; 6) shock; and 7) complicated with other organ failure requiring ICU care.

### 2.2 Nucleic acid testing

A real-time reverse transcription-polymerase chain reaction (RT-PCR) assay was performed to detect the SARS-CoV-2 of nasal swab samples with AutraMic mini4800 Plus equipment. Liferiver (Shanghai ZJ Bio-Tech Co., Ltd.) A novel coronavirus 2019-nCoV nucleic acid detection kit was used. The Ct values of ORF1ab, the nucleocapsid protein (N) and the E gene were obtained. Most inpatients underwent nucleic acid testing every 2 days until meeting discharge criteria, i.e., the Ct values of N gene and ORF1ab gene >35 in two consecutive nucleic acid tests or two consecutive negative results on the nucleic acid test. The minimum Ct values of the ORF gene, N gene and E gene during hospitalization were obtained. Lower Ct values indicated higher SARS-CoV-2 virus copies (26). We applied Ct values to represent the degree of SARS-CoV-2 viral loads.

### 2.3 Burden of comorbidity

The overall burden of comorbidity was assessed by the modified form age-adjusted Charlson comorbidity index (aCCI), which accounts for multiple chronic medical conditions (27, 28). The CCI is the most extensively studied and widely used comorbidity index (29). Data on multiple chronic medical conditions, including myocardial infarction, congestive heart failure, peripheral vascular disease, cerebrovascular accident or transient ischemic attack, dementia, COPD, donnective tissue disease, peptic ulcer disease, liver disease, diabetes mellitus, hemiplegia, moderate to severe chronic kidney disease, solid tumor, leukemia, lymphoma and AIDS were retrospectively collected from electronic medical record. ACCI score was calculated for each patient by using a freely accessible online calculator (https://www.mdcalc.com/charlson-comorbidity-index-cci#use-cases). The mean value of the aCCI score was 4 points. Patients were divided into two groups according to the mean value of the aCCI score. ACCI score >4 point was defined as high burden of comorbidity, and others were defined as low burden of comorbidity.

### 2.4 Statistical analysis

Categorical variables were presented as n (%).Ct values were described by median (IQR). Mean ± SD was calculated for inflammatory indicators. Pearson’s Chi-squared test was performed for categorical variables. The differences in inflammatory indicators among different groups were analyzed by *t-*test. *Wilcoxon Mann-Whitney rank test* was applied to compare the Ct values among different groups. The linear regression models were performed to investigate the associations between inflammatory indicators, the overall burden of comorbidity, and Ct values of ORF gene (the minimum Ct values of the ORF gene during hospitalization). Coefficient values (*β*) and 95% confidence intervals (95% CIs) were calculated.

In order to assess the mediation effects of inflammatory indicators on the associations between the overall burden of comorbidity and Ct values (the minimum Ct values of the ORF gene during hospitalization), a PROCESS model of mediation analysis with R/bruceR package was conducted. The confidence intervals (CIs) of effect estimates were calculated with the bootstrap method. Variations in Ct value over time were visualized by fitting smooth lines using a loess method. Statistical analyses were performed using the R-4.1.2 software. Two-sided test with *P* < 0.05 indicating statistical significance was used.

## 3 Results

### 3.1 Differential associations of Ct values with the overall burden of comorbidity

A total of 767 COVID-19 patients with a mean age of 78.5 years old were included. Among these, 57.2% were female patients, and < 30% received the COVID-19 vaccine. There were 35 severe COVID-19 patients (4.6%). The distributions of different characteristics according to the overall burden of comorbidity were shown in **Table 1**. The proportion of patients with a high burden of comorbidity (9.2%) in the severe group was significantly higher than that of patients with a low burden of comorbidity (1.9%), and the difference was statistically significant (*P*<0.05). In addition, patients with a high burden of comorbidity had a lower rate of COVID-19 vaccine compared with the low comorbidity burden group (*P* < 0.05). It was also found that patients with a high burden of comorbidity had significantly higher levels of white blood cells, neutrophil count, C-reactive protein, and procalcitonin compared to patients with a low burden of comorbidity (all *P* < 0.05). Also, a significantly lower lymphocyte count was observed among patients with a low burden of comorbidity (*P* = 0.022).

**Table 1:** Characteristics of COVID-19 patients according to the overall burden of comorbidity .

Variables	Overall (n=767)	Low burden of comorbidity (n=285)	High burden of comorbidity (n=482)	P value
Gender				
Male	328 (42.8)	199 (41.3)	129 (45.3)	0.317
Female	439 (57.2)	283 (58.7)	156 (54.7)	
Vaccination				
No	499 (70.2)	272 (59.9)	227 (88.3)	**<0.001**
Yes	212 (29.8)	182 (40.1)	30 (11.7)	
Group				
Non-severe group	730 (95.4)	472 (98.1)	258 (90.8)	**<0.001**
Severe group	35 (4.6)	9 (1.9)	26 (9.2)	
**Minimum Ct value of ORF gene**	25.73 (11.25)	26.58 (11.5)	24.81 (10.49)	**0.005**
**Minimum Ct value of N gene**	25.39 (12.43)	26.14 (12.7)	24.39 (11.77)	**0.013**
**Minimum Ct value of E gene**	25.71 (11.24)	26.10 (11.34)	24.89 (11.05)	0.065
**WBC(x 10^9^/L)**	6.15 (3.32)	5.61 (2.07)	6.95 (4.47)	**<0.001**
**N(x 10^9^/L)**	4.11 (4.08)	3.67 (4.23)	4.76 (3.76)	**0.002**
**L(x 10^9^/L)**	1.49 (1.19)	1.59 (1.00)	1.35 (1.41)	**0.022**
**CRP(mg/L)**	26.21 (47.56)	14.18 (30.71)	43.36 (60.46)	**<0.001**
**PCT(ng/mL)**	0.74 (4.08)	0.15 (0.59)	1.39 (5.82)	**0.025**

WBC, White blood cell count; N, Neutrophil count; L, Lymphocyte count; CRP, C-reactive protein; PCT, Procalcitonin. The bold values mean statistical significance.

**Figure 1:**
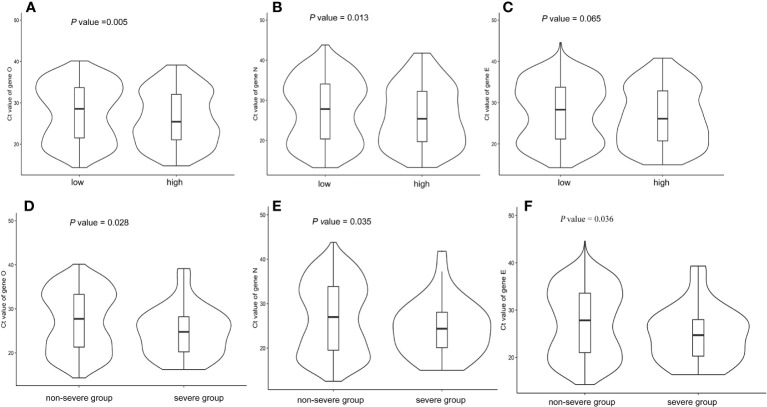
Boxviolin plots of Ct values. **(A–C)**: Distributions of Ct values for target genes according to the burden of comorbidity. **(D–F)**: Distributions of Ct values for target genes according to disease severity. *P* values were calculated by a two-tailed Mann–Whitney test between groups.

The median, minimum Ct value of the ORF gene during hospitalization in patients with a high burden of comorbidity was 24.81, which was significantly lower than that in subjects with a low burden of comorbidity (median, 26.58, *P* < 0.05). Similarly, the Ct values of the N gene and E gene were lower in the high burden of the comorbidity group compared to the low burden of the comorbidity group. **Figures 1A–C** showed the distributions of Ct values according to the overall burden of comorbidity. COVID-19 patients with a high burden of comorbidity were more likely to have lower Ct values (*P* < 0.05), indicating higher viral loads. It was also found that severe COVID-19 patients had lower Ct values (**Figures 1D–F**).

### 3.2 Differential association of Ct values with disease severity depending on patient’s overall comorbidity burden

**Figure 2:**
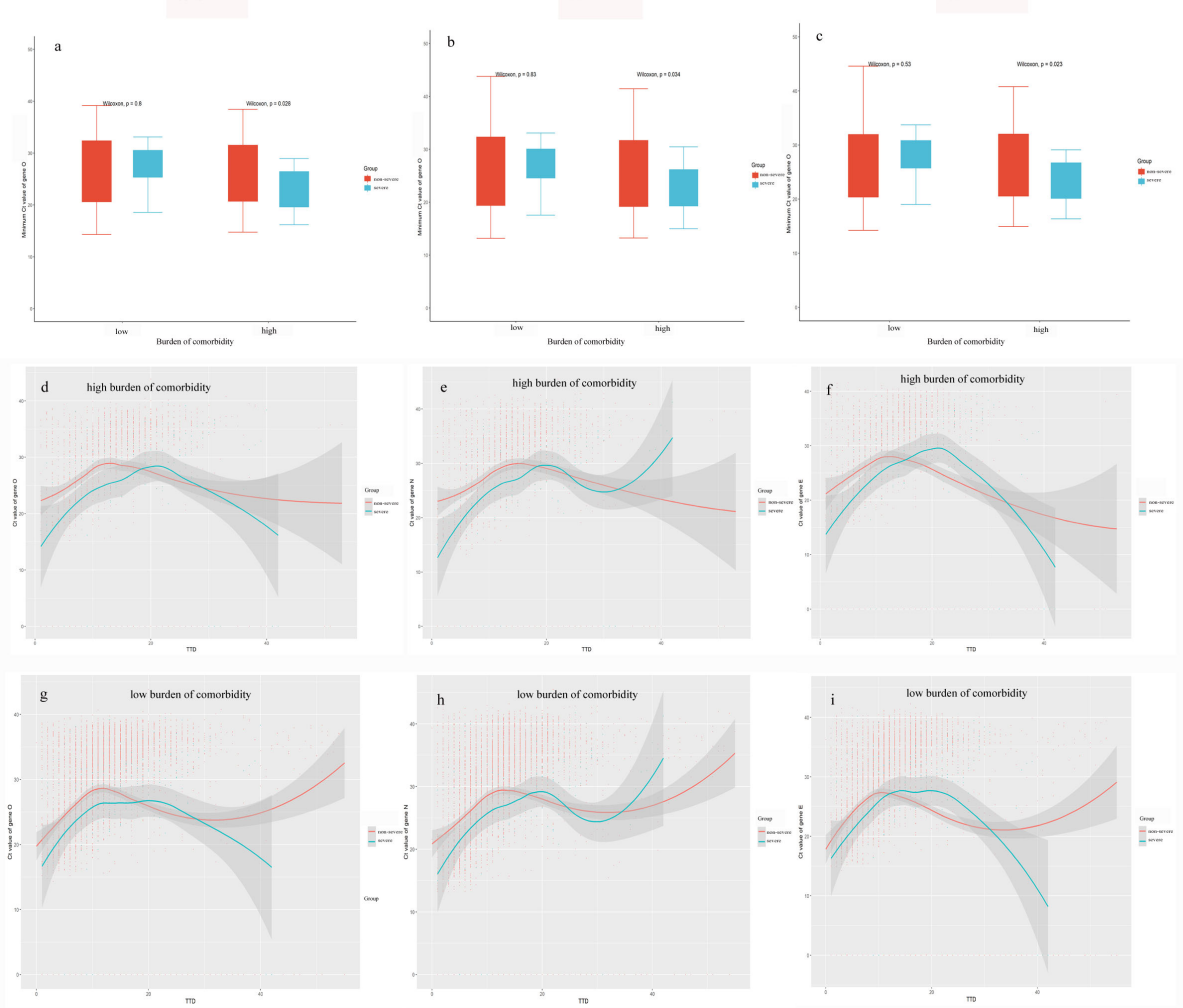
Kinetic changes of Ct values according to disease severity. **(A–C)**: Distributions between disease severity and Ct values for target genes in high and low comorbidity burden subgroups. **(C–E)**: Kinetic changes of Ct values for target genes in patients with a high burden of comorbidity from severe and non-severe groups. **(F–I)**: Kinetic changes of Ct values for target genes in patients with a low burden of comorbidity from severe and non-severe groups. The blue and red lines showed the trend in viral loads of severe and non-severe groups, respectively, using curve fit with non-linear regression with 95% confidence intervals (shaded color) from the regression line. TTD: Times to detection of nucleic acid testing.

A subgroup analysis was conducted to confirm whether the lower Ct values in patients with a high burden of comorbidity might be due to the increased prevalence of severe COVID-19 patients compared to patients with a low burden of comorbidity (**Figure 2**). Our results showed that the associations between Ct values and disease severity depended on the burden of comorbidity, wherein disease severity was significantly related to lower Ct values only in patients with a high burden of comorbidity (*P* < 0.05) but not in patients with a low burden of comorbidity. Ct values dynamics of patients also showed severe patients had lower Ct values during the first 20 days recovery process compared with non-severe patients in the subgroup with the high comorbidity burden (**Figures 2D–F**). Also, this phenomenon was not obvious in the subgroup with a low burden of comorbidity. These results suggested that viral clearance in COVID-19 patients might be differently regulated according to the burden of comorbidity.

### 3.3 Mediation effects of inflammatory markers on the association between the overall burden of comorbidity and Ct values

Our results showed that a high burden of comorbidity was significantly associated with higher inflammatory response, including white blood cell count (*β*: 1.23, 95% CI: 0.59, 1.86), neutrophil count (*β*: 1.21, 95% CI: 0.66, 1.76), and C-reactive protein (*β*: 20.32, 95% CI: 11.04, 29.6) (**Table 2**). Furthermore, a linear regression model was applied to explore associations between inflammatory indicators and Ct values (**Table 3**). Higher C-reactive protein was associated with lower Ct values with a coefficient value of -0.02 (95%CI: -0.03,-0.01, *P* = 0.0223). White blood cell and neutrophil count were positively associated with Ct values (*β* [95% CI]: 0.28[0.12-0.45] and 0.20[0.07,0.33], respectively). Meanwhile, a high burden of comorbidity was significantly associated with lower Ct values (*β* [95% CI]: -1.08 [-2.09,-0.06]).

**Table 2:** Associations between inflammatory indicators and the burden of comorbidity.

Inflammatory Indicators	*β*[95%CI]^a^	*P* value
**WBC**	**1.23 [0.59,1.86]**	**<0.001**
**N**	**1.21 [0.66,1.76]**	**<0.001**
**L**	-0.14 [-0.38,0.10]	0.2609
**CRP**	**20.32 [11.04,29.6]**	**<0.001**
**PCT**	0.82 [-0.16,1.81]	0.0988

a Inflammatory indicators as the response variable and comorbidity burden group as the predictor variable in linear regression models. Models adjusted for gender, vaccination, and disease severity. The bold values mean statistical significance.

WBC, White blood cell count; N, Neutrophil count; L, Lymphocyte count; CRP, C-reactive protein; PCT, Procalcitonin.

**Table 3:** Associations between inflammatory indicators and Ct values.

Variables	β[95%CI]^a^	*P* value
**burden of comorbidity**		
** Low**	**ref**	**0.037**
**High**	**-1.08 [-2.09,-0.06]**	
**WBC**	**0.28 [0.12,0.45]**	**<0.001**
**N**	**0.20 [0.07,0.33]**	**0.003**
**L**	0.40 [-0.05,0.85]	0.079
**CRP**	**-0.02 [-0.03,-0.01]**	**0.022**
**PCT**	-0.12 [-0.37,0.14]	0.369

a adjusted for gender, vaccination, and disease severity. The bold values mean statistical significance.

WBC, White blood cell count; N, Neutrophil count; L, Lymphocyte count; CRP, C-reactive protein; PCT, Procalcitonin.

After a detailed exploration of the associations among the overall burden of comorbidity, Ct values, and inflammatory indicators, we assumed that the inflammatory indicators mediate the association between the burden of comorbidity and Ct values, which was subsequently confirmed through a mediation analysis (**Table 4**). The total effect estimates of white blood cell and c-reactive protein on Ct values were -1.289 (95% CI: -2.393, -0.069) and -1.630 (95% CI:-2.832, -0.299), respectively. The mediation effect of white blood cells on the association between the comorbidity burden group and Ct values was found, with the indirect effect estimates of 0.381 (29.56% of the total effect size, 95% CI: 0.166, 0.632, *P* < 0.001). Similarly, C-reactive protein was found to significantly mediate the relationship between the burden of comorbidity and Ct values (18.13% of the total effect size, *β*= -0.307, 95% CI: -0.645, -0.064, *P* = 0.034). No mediation effects were observed for the neutrophil count, lymphocyte count, and procalcitonin.

**Table 4:** Mediation effect estimates of inflammatory indicators on the association between the burden of comorbidity and Ct values.

Inflammatory Indicators	Effect estimates	Effect	95% CI	P value	Prop.Mediated
**WBC**	**Indirect**	0.381	0.166, 0.632	**0.001**	**29.56%**
	**Direct**	-1.670	-2.765, -0.450	**0.007**	
	**Total**	-1.289	-2.393, -0.069	**0.037**	
**N**	**Indirect**	0.192	-0.017, 0.497	0.125	14.84%
	**Direct**	-1.486	-2.769, -0.270	**0.015**	
	**Total**	-1.294	-2.530, -0.069	**0.036**	
**L**	**Indirect**	-0.053	-0.662, 0.020	0.752	4.12%
	**Direct**	-1.232	-2.407, 0.076	**0.056**	
	**Total**	-1.285	-2.470, -0.004	**0.039**	
**CRP**	**Indirect**	-0.307	-0.645, -0.064	**0.034**	**18.83%**
	**Direct**	-1.324	-2.577, 0.024	**0.047**	
	**Total**	-1.630	-2.832, -0.299	**0.013**	
**PCT**	**Indirect**	-0.094	-0.256, 0.075	0.259	6.20%
	**Direct**	-1.422	-3.248, 0.398	0.120	
	**Total**	-1.516	-3.360, 0.299	0.094	

WBC, White blood cell count; N, Neutrophil count; L, Lymphocyte count; CRP, C-reactive protein; PCT, Procalcitonin. The bold values mean statistical significance.

## 4 Discussion

The present study aimed to explore the effect of the overall burden of comorbidity and inflammatory indicators on Ct values among elderly patients with COVID-19. Our findings highlighted that inflammation-mediated the relationship of comorbidity burden with Ct values. It was also found that Ct values were associated with disease severity depending on patients’ comorbidity burden. To the best of our knowledge, this is the first study that specifically aimed to identify the mediation effect of inflammatory indicators on the relationship between comorbidity burden and Ct values among older patients with COVID-19. Most previous studies solely focused on the associations between the single medical chronic disease and inflammatory indicators with Ct values (16, 21, 24).

The Omicron variant is characterized by immune evasion (30). In the present study, a high burden of comorbidity was associated with lower Ct values (high viral loads) in older patients with Omicron infection. This relationship was also detected in patients with earlier variant infection (9, 17). It is worth noting that compared to the earlier variant, the rate of severe Omicron infections increased among the elderly (31). The low Ct values in the elderly with a high burden of comorbidity may contribute to the disease severity. Additionally, the results highlighted that the overall burden of comorbidity assessed by the age-adjusted Charlson comorbidity index was an independent risk factor for the Ct values. It has been reported that the Charlson comorbidity index predicted poor clinical outcomes and mortality in COVID-19 patients (7, 32). Therefore the Charlson Comorbidity index might contribute to the management of the older patients with COVID-19.

The underlying mechanism of low Ct values in older COVID-19 patients with a high burden of comorbidity remains unclear. We found that a high burden of comorbidity in older patients was associated with enhanced inflammatory responses in plasma with elevated white blood cell count, neutrophil count, and C-reactive protein compared with subjects with a low burden of comorbidity. Furthermore, serum levels of inflammatory indicators were related to Ct values, which was consistent with previous studies (24, 25, 33). Based on current results, it could be hypothesized that inflammatory indicators might contribute to the association between the burden of comorbidity and Ct values. Therefore, we conducted a causal mediation analysis. Finally, we confirmed that white blood cells and C-reactive protein significantly mediated the relationship between the burden of comorbidity and Ct values by 29.56% and 18.13% of the total effect size, respectively. These results were unsurprising as age-related diseases share inflammatory pathogenesis and age-related decline and dysregulation of immune function (34–36). Also, the degree of immune dysfunction correlates with disease severity (37, 38). Previous studies have reported that white blood cells and C-reactive protein are early indicators of progression to serious disease and in-hospital mortality in COVID-19 patients (39–42). Chen et al. also proposed an immune hypothesis for the COVID-19 vulnerability of older adults (34), which was further supported by our findings. In addition, Padilla et al. reported that remdesivir combined with immunomodulatory therapy had a better effect in patients with Ct values < 25 (43). Also, our findings might contribute to the elucidation of the underlying mechanism. Antiviral treatments combined with immunomodulatory therapy might be particularly helpful for the elderly patients with COVID-19 and a high burden of comorbidity.

The presence of a mediator involves a causal pathway between exposure and outcome (44). Mediation analysis is widely used to explore and evaluate biological mechanisms and unknown biological pathways (45–47). The criteria for certain factors to be regarded as a mediator is that exposure should have a statistically significant association with mediator, and that mediator should also have a statistically significant association with outcome (48). In the current study, we found that the burden of comorbidity was significantly associated with inflammatory indicators. Also, we observed a significant relationship between serum levels of inflammatory indicators and Ct values. Thereby, we conducted a causal mediation analysis, where Ct values, used as an outcome variable, were regressed on inflammatory indicators (mediator variable) and burden of comorbidity (independent variable). Our results supported the partial mediation of inflammation on the association between the burden of comorbidity and Ct values among the elderly.

The latest systematic review reported an inconclusive relationship between COVID‐19 severity and viral loads (11). We observed that the associations between Ct values and disease severity depended on the overall burden of comorbidity, wherein disease severity was significantly related to lower Ct values only in patients with a high burden of comorbidity but not in patients with a low burden of comorbidity. Moreover, we noted that viral clearance was delayed in patients with COVID-19 and a high burden of comorbidity compared to patients with a low burden of comorbidity. The low Ct values (higher viral loads) in older patients with a high burden of comorbidity may explain a potential mechanism underlying the relationship between COVID‐19 viral loads and disease severity.

The present study has some limitations. First, we did not have quantitative viral loads. However, previous studies reported that Ct values were positively associated with viral loads (26). Lower Ct values indicate higher viral loads. Second, some inflammatory markers associated with critical cases of COVID-19, such as IL-6, interleukin (IL)-1β, and TNF-α, were not included (39). Third, we did not have the information about treatments that would allow us to assess the effect of antiviral treatments and immunomodulatory therapy on COVID-19 viral loads. These issues should be addressed by further studies.

In China, there were about 264 million individuals aged≥60 years old in 2020, accounting for 18.70% of the total population (49). If a large number of elderly became infected with SARS-CoV-2 in the future, this would pose a substantial challenge; thus, greater focus should be placed on the elderly with a high burden of comorbidity. In this study, we revealed that a high overall comorbidity burden in older patients with COVID-19 was associated with lower Ct values, partly mediated by inflammation. Moreover, we found that the differential association of Ct values with disease severity among the elderly depended on patient’s overall comorbidity burden. These conclusions have relevant implications for combined immunomodulatory therapies for older patients with COVID-19, which might contribute to effectively reducing the progression to serious disease, especially for the elderly with high burden of comorbidity.

## Data availability statement

The datasets presented in this study can be found in online repositories. The names of the repository/repositories and accession number(s) can be found below: https://github.com/wangmeixia223542/original-data.git.

## Ethics statement

The studies involving human participants were reviewed and approved by the Ethical Committee of Zhongshan Hospital, Fudan University. Written informed consent for participation was not required for this study in accordance with the national legislation and the institutional requirements.

## Author contributions

MW, HM, CS and JP contributed to the study conception and design. Material preparation, data collection and analysis were performed by MW, NL, QS, WS, TH, JL and WJ. MW, HM, XG and BH drafted manuscript. All authors critically reviewed the manuscript and approved the final version.
